# Instantaneous CO_2_ emission modelling for a Euro 6 start-stop vehicle based on portable emission measurement system data and artificial intelligence methods

**DOI:** 10.1007/s11356-023-31022-5

**Published:** 2023-12-29

**Authors:** Maksymilian Mądziel

**Affiliations:** Faculty of Mechanical Engineering and Aeronautics, Rzeszow University of Technology, 35-959, Rzeszow, Poland

**Keywords:** Vehicle emission, Exhaust measurement, Air pollution, CO_2_, Portable emission measurement system, Machine learning

## Abstract

One of the increasingly common methods to counteract the increased fuel consumption of vehicles is start-stop technology. This paper introduces a methodology which presents the process of measuring and creating a computational model of CO_2_ emissions using artificial intelligence techniques for a vehicle equipped with start-stop technology. The method requires only measurement data of velocity, acceleration of vehicle, and gradient of road to predict the emission of CO_2_. In this paper, three methods of machine learning techniques were analyzed, while the best prediction results are shown by the gradient boosting method. For the developed models, the results were validated using the coefficient of determination, the mean squared error, and based on visual evaluation of residual and instantaneous emission plots and CO_2_ emission maps. The developed models present a novel methodology and can be used for microscale environmental analysis.

## Introduction

Transportation is a major sector of the economy that causes environmental pollution and climate change. Emissions from transportation, mainly road transport, contribute significantly to the amount of greenhouse gases in the atmosphere (Wang et al. 2020c; Giannakis et al. 2020). Automotive is the second largest CO_2_ producing sector with a global total share of approximately 1/4 of total emissions (Rubio et al. 2020). The health effects associated with road transport-related pollution have led to stricter environmental regulations for vehicle exhaust gases (Grigoratos et al. 2019; Giechaskiel et al. 2019). As a result, in 1992, the European Union introduced emission standards for the maximum permitted amounts of harmful components of exhaust gases (Varella et al. 2019). The permissible amounts and standards are constantly evolving, and work is currently underway to publish Euro 7 standards, which, along with the CO_2_ emission standards regulations, will set a new direction for future vehicle technology (Selleri et al. 2022). The new regulations, in line with the European Green Deal, aim to make Europe a climate-neutral continent by 2050 (Montanarella and Panagos 2021; Sikora 2021). Moreover, several Asian and Latin American countries are currently applying, to some extent, the European emission requirement (Bharj et al. 2019).

The amount of emissions from exhaust components, including CO_2_, is estimated based on vehicle test procedures — WLTP (Worldwide Harmonized Light-Duty Vehicles Test Procedure), supplemented by road tests (Pavlovic et al. 2018; DiPierro et al. 2019). Many researchers study real-world emissions by performing various driving cycles on selected routes, not necessarily based on homologation procedures (Weller et al. 2019; Gao et al. 2022). The estimated emissions are for a specific vehicle model, but the challenge is to estimate emissions for all vehicle traffic, which consists of many different types of vehicles that run on different fuels and have different emissions-reducing technologies. One such technology is the start-stop system.

The start-stop technologies used by vehicle manufacturers are low-cost solutions that rely on the internal combustion engine to automatically shut down when the vehicle is stationary and restart at the driver's request (Santos et al. 2020). Technology is particularly useful in city center, where the vehicle performs many stop-and-go operations and spends a lot of time in traffic congestion (Wang et al. 2020a). This reduces the fuel consumption of the idle phase of the engine and, consequently, also reduces total exhaust emissions (Zhu et al. 2022).

Proper estimation of vehicle emissions is a major challenge. Measurement of emissions from vehicular traffic is even more problematic. A method that makes it possible to estimate emissions for vehicular traffic is to use, for example, models that allow emissions to be calculated (Perugu 2019; Kumar et al. 2021). A general division of emission models includes macro- and microscales (Hulagu and Celikoglu 2021). The macroscale allows emission estimates based on fewer data, mainly the technical data of the vehicle and its average velocity (Rodriguez-Rey et al. 2021). Models that allow for more accurate estimates are microscale models that need road data such as velocity, acceleration, and road gradient as a function of time (Lejri et al. 2018). Based on such calculations, it is possible to estimate emissions for the entire route, determine emission maps, and average emission factors, which, for example, can be related to emission standards, such as the actual applicable Euro 6 standard. Accurate measurement and estimation of vehicle emissions are important in the context of conducting, for example, environmental analyses of the impact of harmful transportation on the health of pedestrians traveling along major urban arteries (Quaassdorff et al. 2016; Borge et al. 2016). Creating emission maps based on emission models provides information on where and in what quantities harmful components of exhaust gases accumulate (Sanchez et al. 2017). The modeling approach is even more important because we can make models of future road investments and analyze them even before making a decision to build a particular type of road solution.

Accurate measurement of CO_2_ emissions is also very important, as this component is considered to be the largest contributor to global warming (Campisi et al. 2021). Carbon dioxide has the largest share of all greenhouse gases (excluding water vapor) in their total amount and is the main source of global warming (Yoro and Daramola 2020; Franta 2018). Total carbon dioxide emissions in 2001 reached 34 million tons. Currently, the world’s anthropogenic CO_2_ emissions reach about 38 billion tons per year (Anenberg et al. 2019). Unless radical policies are implemented to reduce this emission, annual CO_2_ emissions are estimated to increase to 41 billion tons by 2025 (Mikhaylov et al. 2020).

A review of articles in the field of using artificial intelligence for emission prediction shows that the base of such papers is small. Publication data from the Web of Science Core collection show that there are only 82 papers that present the results of using AI in emissions modeling. More entries are found for the keywords emissions modeling and machine learning. One such paper is (Hoang et al. 2021) in which the authors use the artificial neural network (ANN) method to predict emissions from biodiesel-fueled diesel engines. Another example is the work (Le Cornec et al. 2020), in which the authors create emission models with a particular focus on NO_x_ emissions using machine learning techniques. In this work, models using neural network multilayer perceptron (MLP) techniques showed the best predictive capabilities for NO_x_ emission estimates. Another work using artificial intelligence techniques is (Azeez et al. 2019), where the authors created a model of CO emissions based on traffic volume data with the use of ANN. The resulting model achieved a prediction accuracy of about 80.6%. Computational models also apply to the topic of electric vehicles; an example is the work (Zhu et al. 2019), in which the authors made an accurate model of the charge load of EV using the gated recurrent unit (GRU) method. However, there is a lack of work dedicated to predicting emissions for vehicles with start-stop technology. Choosing the right machine learning method for CO_2_ prediction is crucial, as not all of them will be able to restate zero emissions during most vehicle stops.

**Fig. 1:**
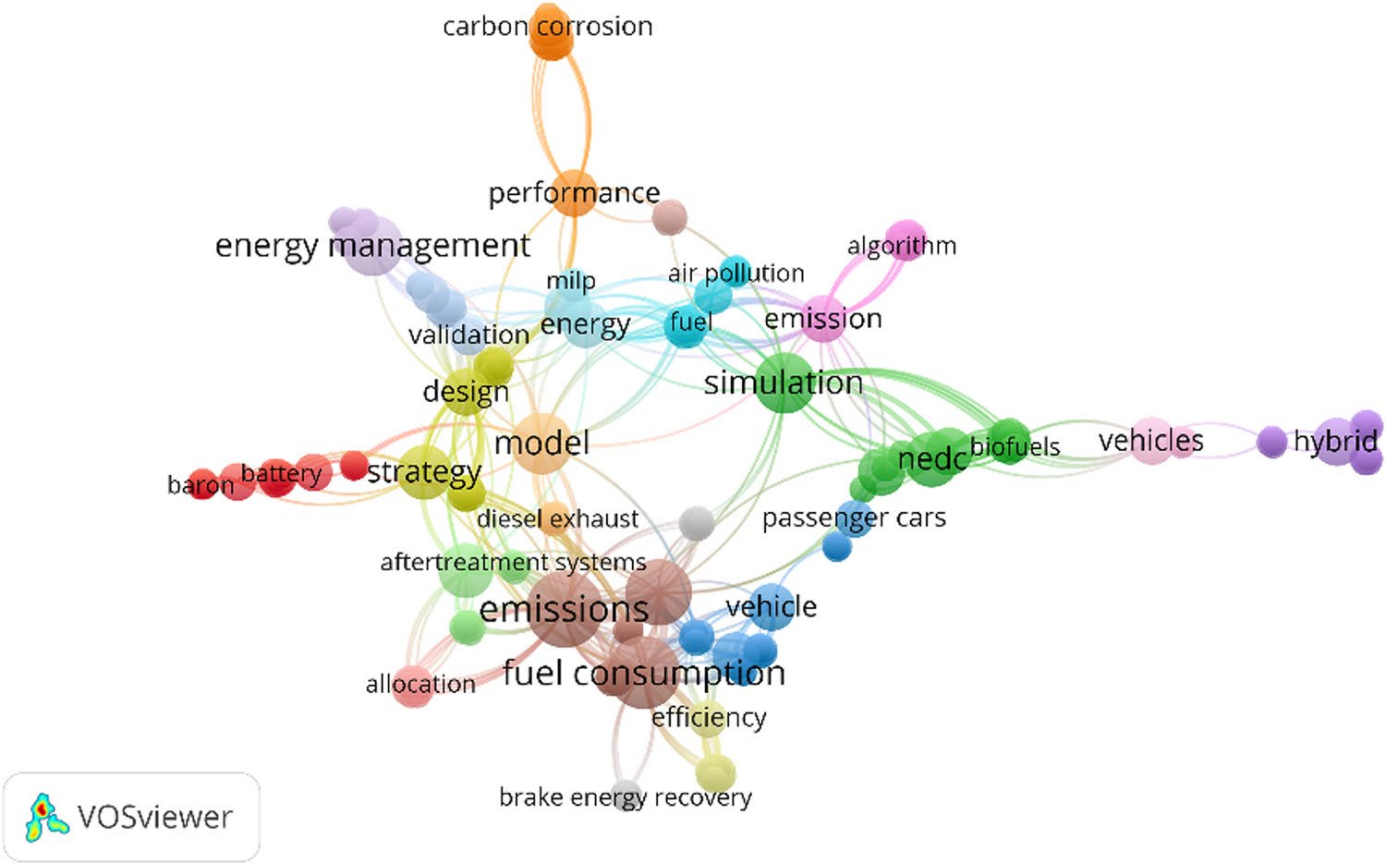
VOSviewer network visualization based on Web of Science article review for the research topic keywords: emission, model, and start-stop

Publication data in the Web of Science Core collection show 68 results of papers for the keywords emission, model, and start-stop, while none of these papers after analysis of abstracts addresses the scope of emission modeling for start-stop technology. Therefore, this paper will be one of the first to address the scope of emissions modeling for vehicles with start-stop systems. For the above keywords, a graphic was generated using the VOSviewer software (Shah et al. 2019) showing the link of all keywords in these articles to each other (Fig. 1). From it, one can see a strong connection between the topics of emissions, fuel consumption, model, and vehicles, with simulation also present in close proximity. No start-stop keyword is found in this graphic, further confirming the lack of developed models of this type.

In view of items from the analysis of commonly available literature, the purpose of this work will be to perform a research methodology to obtain a correct model to estimate CO_2_ emissions for a vehicle with start-stop technology using artificial intelligence methods. In the work, a microscale emission model is created. It is based on real data measured by the portable emission measurement system (PEMS). Furthermore, the data were measured via an OBD II (on-board diagnostic) interface. Data on speed, acceleration, vehicle coordinates, and actual CO_2_ emissions were selected to create a CO_2_ emissions model for start-stop technology. The most important and desirable feature of the obtained model is the CO_2_ prediction capabilities for a zero value of these emissions under start-stop vehicle operations. The obtained model, a modern and accurate model using artificial intelligence techniques, can be used for environmental analysis by, for example, road managers or city authorities as a component.

## Methods

**Fig. 2:**
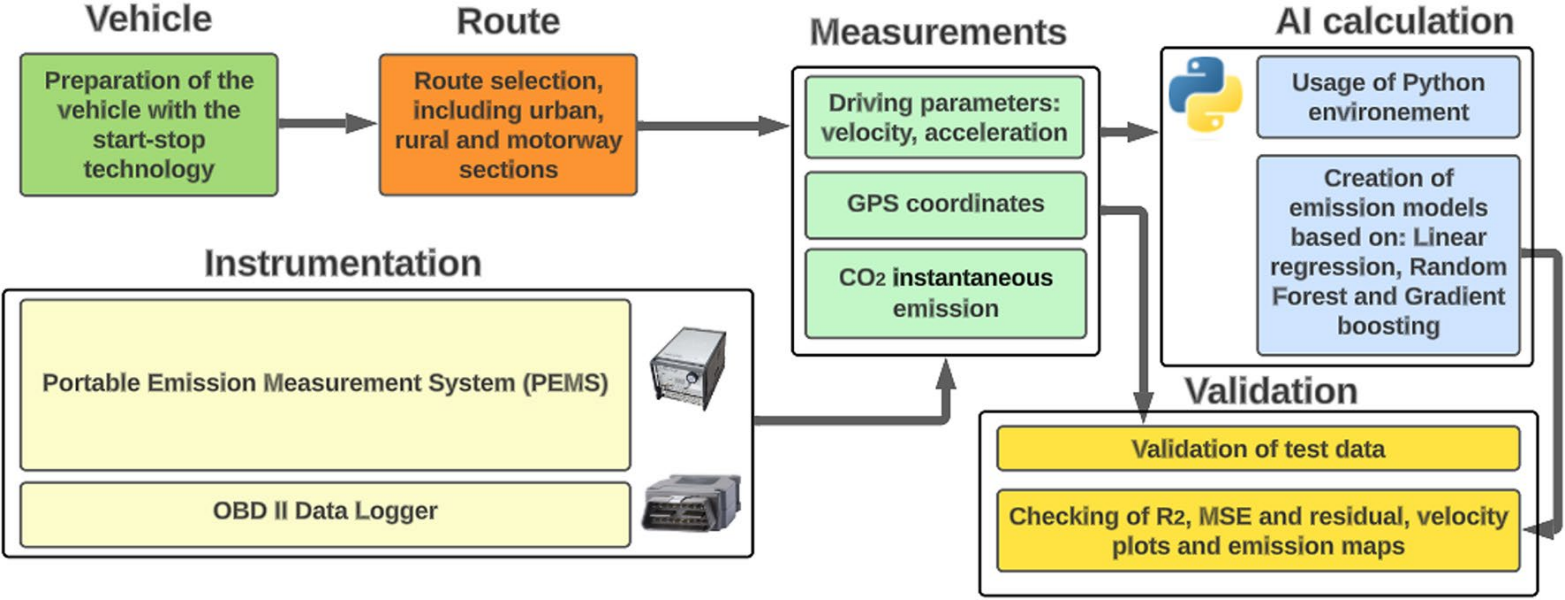
Basic logic and scheme of the performed research

The general structure of the work is shown in Fig. 2. The study can be divided into three main stages. In the first part, it was important to properly prepare the vehicle and install the PEMS system. The PEMS system was properly calibrated against the reference gases, and an OBD II interface was connected to the vehicle to record vehicle parameters. In this part, it was also necessary to prepare a driving route that would take into account a wide range of velocity and driving characteristics for different engine loads. In the second step, road tests were conducted on the selected parts of city roads to collect enough data. The focus was on measuring the following data, which were then used to construct computational models using artificial intelligence techniques, namely driving velocity, vehicle acceleration, road gradient, and CO_2_ emissions. This data set, after initial filtering of the results, was then uploaded to a repository and transferred to Google Colab. Google Colab is a Jupyter environment provided and operated by Google with the ability to work with CPUs, GPUs, and even TPUs (Carneiro et al. 2018; Bisong 2019). The operation of coding the models and creating the associated visualizations was done in the Python environment. Python is a general-purpose high-level programming language with an extensive suite of standard libraries whose guiding idea is the readability and clarity of its source code. Its syntax is characterized by clarity and conciseness (Raschka et al. 2020; Hao and Ho 2019). The article describes a data storage approach for model development that combines the capabilities of Google Colab and a GitHub data repository. This strategy provides several advantages, including version control to maintain a comprehensive history of dataset changes, ensuring transparency and traceability throughout the development process. It also facilitates collaborative work among researchers, offering a secure cloud-based environment for model development. Data security is upheld through GitHub's access controls, which restrict data access to authorized individuals. Critically, the methodology allows for ongoing model improvement through data updates. The approach enables automated data pipelines that fetch and preprocess data from the GitHub repository, enabling the real-time integration of new data points. This adaptability enables continual refinement and enhancement of the future model's accuracy. Linear regression, random forest, and gradient boosting methods were selected for calculating computational models. These are methods with different abilities to represent real data. The validation of the models obtained was carried out based on MSE and *R*^2^, as well as visual validation based on emission, maps, instantaneous emission, and residual plots. The methodology developed, with special emphasis on start-stop technology, is one of the first of its kind. However, the main objective of the work was to create a CO_2_ emissions model that gives the most accurate picture of emissions. Creating CO_2_ models for stop-start technology is problematic because this vehicle, unlike a conventional one without this system, does not emit any harmful compounds during most stop-start operations. The use of appropriate computational techniques that can reflect the characteristics of such emissions is needed.

**Fig. 3:**
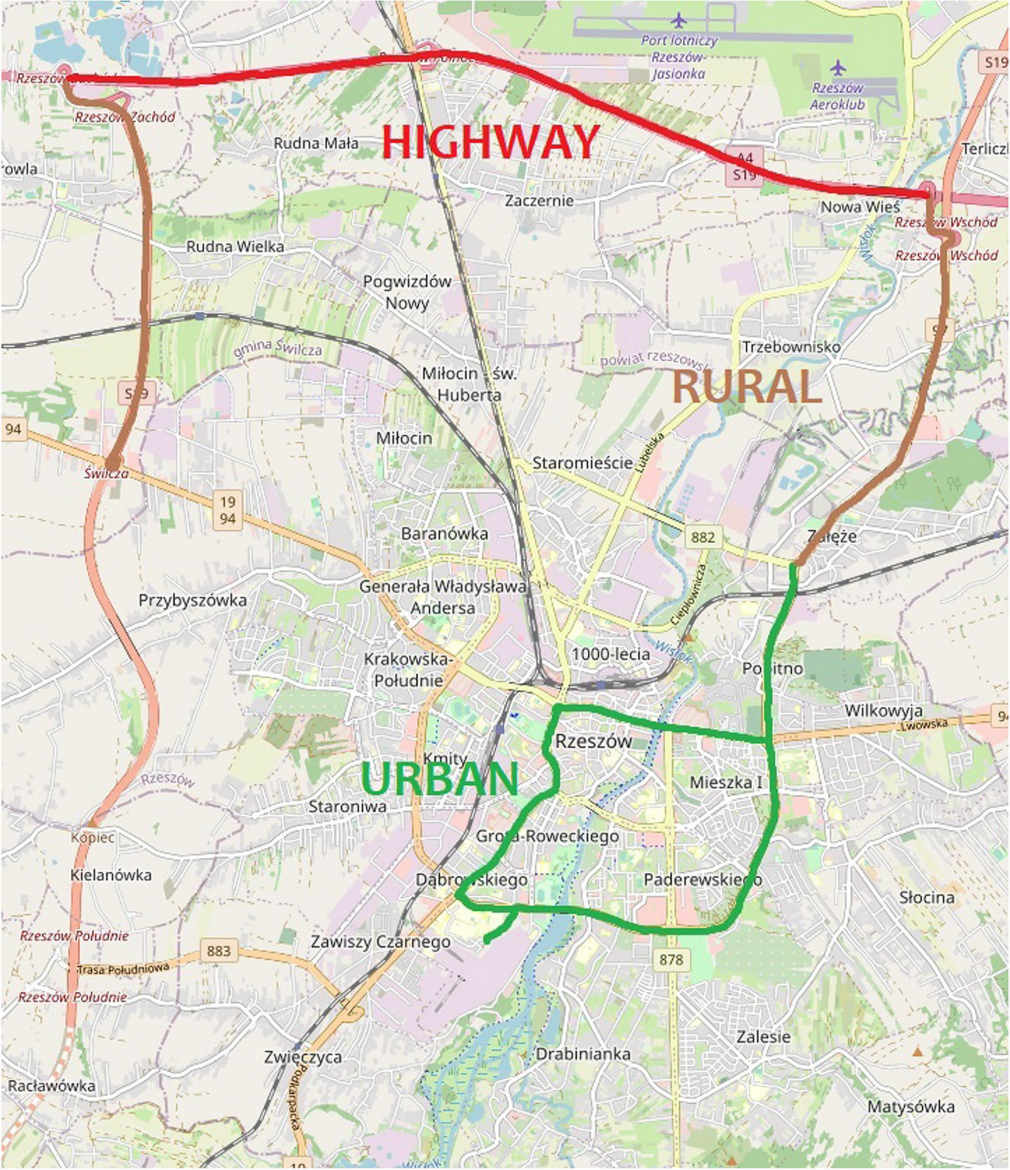
View of the test road with division to the researched part: urban, rural, and highway

The tests were carried out on a Euro 6 compliant vehicle manufactured in 2018. The vehicle has a 1560-dm^3^ diesel engine; the maximum power of the vehicle is 88 kW at 3500 rpm. The vehicle has a manual transmission with a 6-stage gearbox. The vehicle’s fuel injection system is CR (common rail), while the exhaust aftertreatment system is DPF (diesel particulate filter), SCR (selective catalytic reduction), and DOC (diesel oxidation catalysts). The unloaded weight of the vehicle is 1429 kg. The vehicle is equipped with start-stop technology, which allows the engine to temporarily be turned off while at a standstill, such as while waiting for a green light, which is designed to save fuel (Lijewski et al. 2021). This system is extremely useful and has a significant impact on reducing emissions, including CO_2_ under congested conditions (Hao et al. 2020; Deng et al. 2020). Before starting the vehicle road test, the vehicle was checked with the use of stationary exhaust gas analyzers, according to the periodic diagnostic test procedure. For road tests, the route shown in Fig. 3 was selected. The route was driven twice, representing a total of 111 km, and more than 3000 data records were recorded for the model creation with a frequency of 1 s. Such values were sufficient to create an emission model for CO_2_ for the case investigated. The selected parameters of the total distance with division to parts of the route measured using GPS and OBD II are presented in Table 1.

**Table 1:** Selected measurement parameters of the research route

Parameter	Measurements
Total distance (km)	111.4
Distance of the urban part (km)	32.24
Distance of the rural part (km)	39.66
Distance of the highway part (km)	39.5
Average speed (km/h)	44.01
Urban average speed (km/h)	29.77
Rural average speed (km/h)	90.59
Highway average speed (km/h)	110.26

The rationale behind selecting this particular route was based on the need to include a comprehensive representation of the road conditions encountered during the road test. This comprehensive route was deliberately designed to incorporate segments reflecting urban, rural, and highway environments. It is important to note that the primary objective of the road test was not to determine emission factors or adhere strictly to the measurement procedure typically associated with real driving emissions (RDE) tests. Instead, the overarching objective was to accumulate a diverse and extensive dataset, capturing a wide spectrum of velocity and acceleration patterns. Equally vital was the effort to maintain a well-rounded cross section of road gradients throughout the route. This particular parameter, coupled with velocity and acceleration data, constituted the fundamental building blocks for the development of the CO_2_ calculation model, emphasizing the multifaceted nature of the data collection approach.

**Fig. 4:**
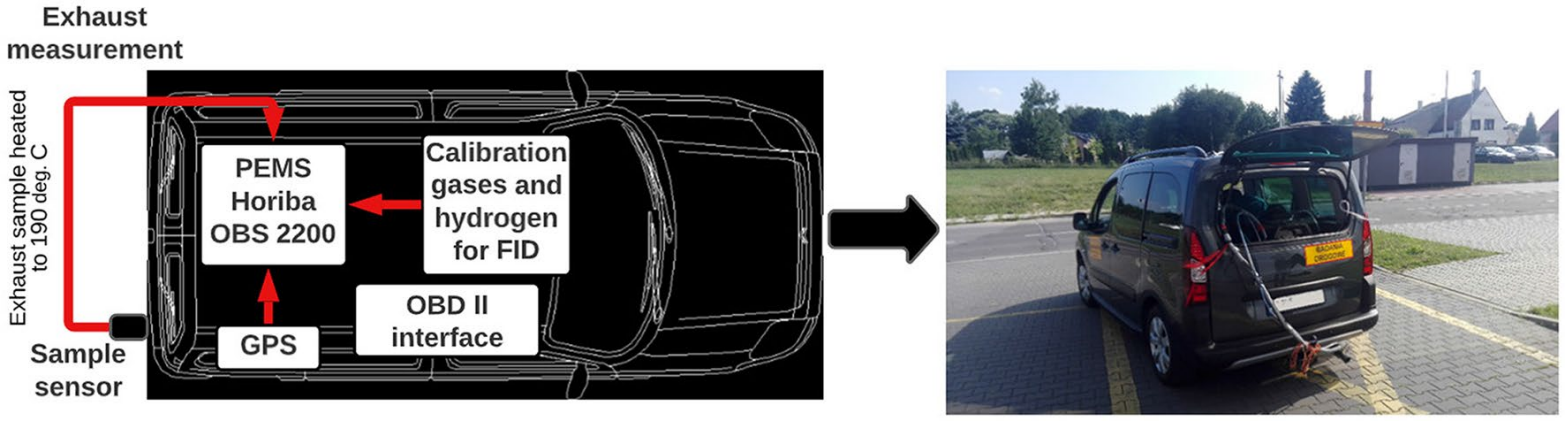
PEMS platform installed in the vehicle in the study, scheme and the real photo

The test vehicle was adapted to connect a PEMS instrumentation, Horiba OBS-2200 (Fig. 4). The PEMS system to measure CO_2_ has a nondispersive infrared (NDIR) spectrometer (Jaworski et al. 2018). The measurement accuracy of the NDIR analyzer for this PEMS system is ± 2.5% (Tipanluisa et al. 2021). A full specification of the accuracy of the measurement and the selected technical parameters of the PEMS is shown in Table 2.

**Table 2:** Selected technical parameters of the PEMS Horiba OBS-2200 system

Parameter	Measurement method	Accuracy
The concentration of exhaust gas components: CO_2_ Sampling frequency	NDIR — nondispersive (infrared), range 0–10% 0.1–1 Hz	± 2.5%
The heating time of the analyzers	Up to 1 h	-
Gas flow	Mass flow rate	In the range of the ± 1.5% of full scale or within ± 2.5% of readings

The PEMS can be installed in the trunk of the vehicle under test, while the flow meter measuring sensors are connected to the exhaust pipe. The exhaust gas sampling tube must be heated to 190 °C to avoid condensation of hydrocarbons (Santos et al. 2021). In addition, ambient temperature and humidity sensors are connected to the system, as well as a GPS transmitter. Vehicle data was recorded in PEMS memory and connected to the OBD II interface, ELM327. Data that were measured included velocity (V), vehicle acceleration (a), fuel consumption, CO_2_ emission, humidity, air temperature, latitude, longitude, and altitude above sea level.

Calculations of CO_2_ emissions from the NDIR detector of the PEMS system were made based on the following Eq. (1):

1 $${{\mathrm{CO}}_{2}}_{\mathrm{MASS}}\left(t\right)={C}_{\mathrm{EXCO}2}\left(t+{DT}_{\mathrm{CO}2}\right)\cdot {M}_{\mathrm{CO}2}\cdot {Q}_{\mathrm{EX}}\left(t\right)\cdot \frac{1}{60}\cdot \frac{1}{100}\cdot \frac{1}{22.415}\cdot \frac{273.15}{293.15}$$

where:

CO_2MASS_(*t*): real-time instantaneous CO_2_ emissions (g/s),
DT_CO2_: analyzer measurement delays relative to flow meter (s),
C_EXCO2_(t + DT_CO2_): concentration of CO_2_ (%),
M_CO2_: molecular weight CO_2_ (g/mol),
Q_EX_(t): flue gas flow rate under standard conditions (293.15 K, 101.3 kPa) at time *t* (l/min).

The data collected were used to create computational models of CO_2_ emissions. The input data were the velocity and acceleration of the vehicle, as well as the road gradient; this set of data can be defined as explanatory variables. The dependent variable, on the other hand, was CO_2_ emissions. The models were developed in Python programming language for computational techniques: linear regression, random forest, and gradient boosting. Such a range of chosen techniques will allow comparison and selection of the best one, which will best reflect CO_2_ emissions for the start-stop technology of the vehicle. The MSE and *R*^2^ indices were used to validate the prepared models. It was also important to perform a visual verification based on actual and modelled emissions graphs, as well as an analysis of emissions maps and residual plots for the models obtained.

## Results

It was important for the development of a CO_2_ emission model for start-stop technology for the vehicle tested that the model prepared could reflect the moments when the vehicle’s engine is turned off, e.g., during momentary stops at junctions. Current microscale models for emission maps and for calculating instantaneous emission values did not allow such results (Eijk et al. 2018; Mądziel and Campisi 2022). Therefore, it is necessary to analyze instantaneous emission plots for CO_2_, emission maps, residual graphs, and validate the models using *R*^2^ and MSE indices.

**Fig. 5:**
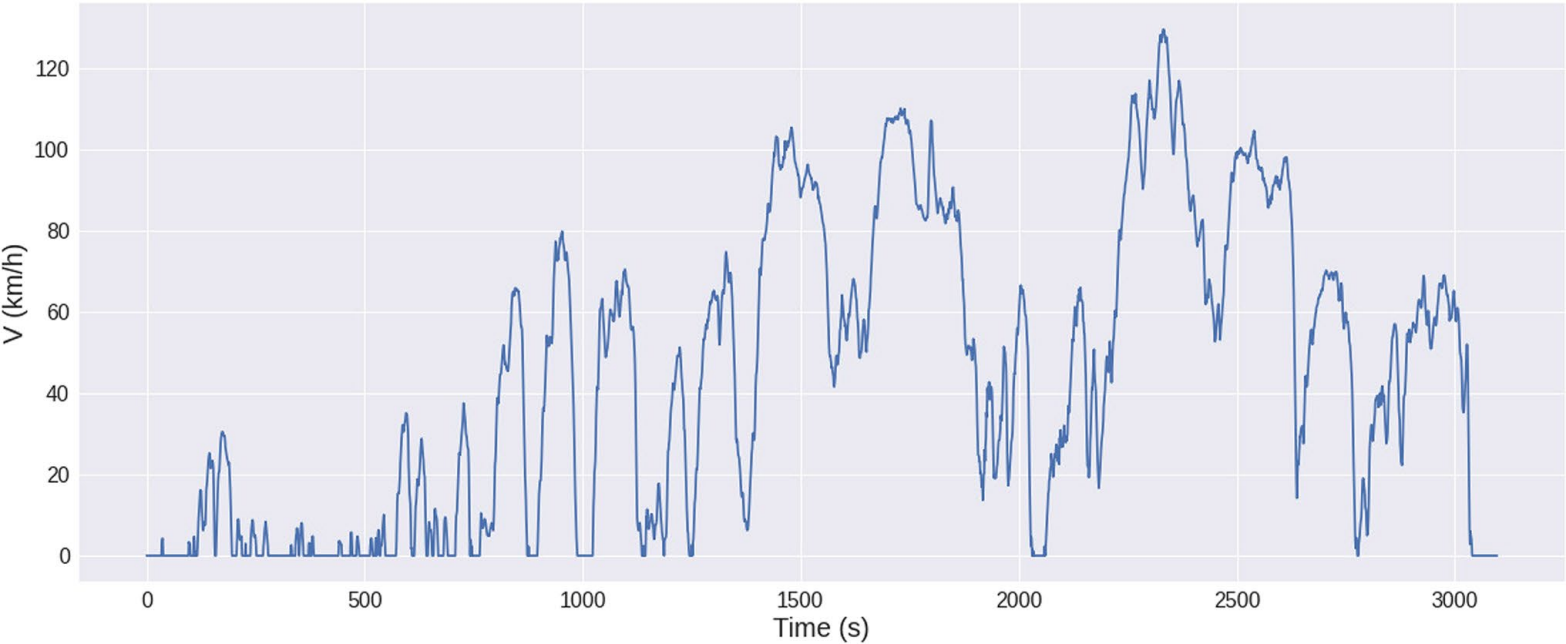
Velocity of the vehicle as an input for the machine learning modeling

The input data for the development of the emission models were the parameters: velocity, acceleration, and road gradient. These data were collected from the PEMS recording and are additionally recorded via OBDII for additional verification of the values obtained. The velocity data for the route tested for the driving cycle are shown in Fig. 5.

The purpose of collecting velocity data was to collect them for values that characterize urban, rural, and highway driving. The appropriate range covered speeds between 0 and 130 km/h. It is important to collect enough data, as the actual portion will be split into 80% as a training set and 20% as a test set. With more data collected, a more accurate model of CO_2_ emissions can be created. However, it should be noted that testing with the PEMS system is relatively expensive, as a large number of calibration gases are required to zero the instrument and prepare it for performing and collecting the correct road test data. Collecting a sufficient amount of data is crucial to the correct implementation of future predictions, as evidenced, for example, by the work of Kan et al. (2018a, b) and Zhang et al. (2022).

**Fig. 6:**
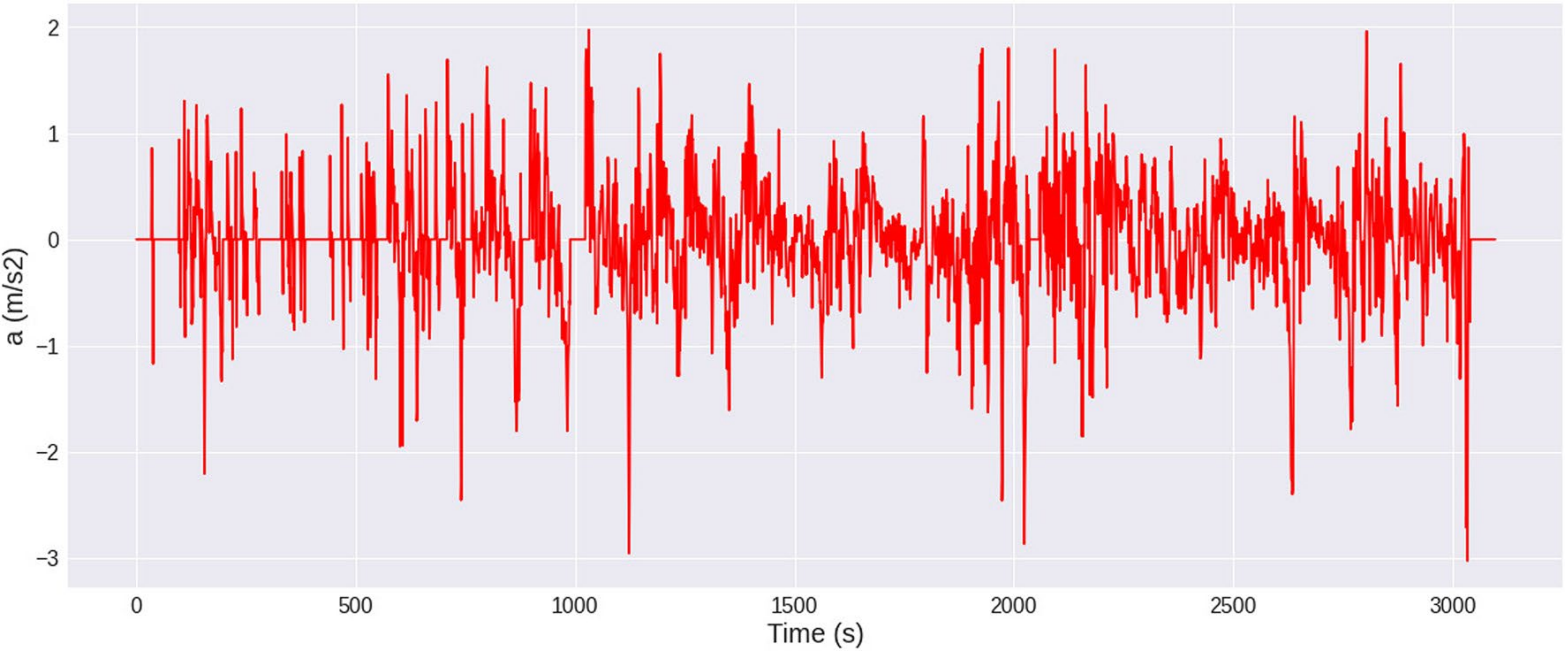
Acceleration (*a*) of the vehicle as an input for the machine learning modeling

Equally important as data collection for velocity is data for vehicle acceleration. Data for an acceleration during the driving cycle are shown in Fig. 6.

Similarly to velocity, the acceleration parameter must be related to different driving characteristics. In urban conditions, acceleration often has to be dynamic, but sometimes in congested conditions, it is somewhat reduced. In rural conditions, acceleration refers to a velocity greater than 60–100 km/h. In general, acceleration is somewhat less dynamic in these ranges due to traffic conditions as well as vehicle capabilities, while driving for different driving styles is relevant here. Acceleration is equally relevant for highway driving, where speeds range from 100 to 130 km/h. The importance of this set for the development of a good model is described, among others, in Zhang et al. (2021a) and Peng et al. (2022).

Another important parameter that provided an input value for the development of CO_2_ emission models using artificial intelligence techniques was the road gradient. The road gradient is a factor that greatly influences the results obtained for CO_2_ emissions. It also forms the input base for the emission models already developed. The importance of this parameter in relation to fuel consumption and emissions is indicated, among others, in the works Rosero et al. (2021) and Liu et al. (2019).

The data for velocity, acceleration, and gradient constituted the so-called independent variable, which served as input data to create a CO_2_ emission model for a vehicle with start-stop technology. Therefore, the CO_2_ data could be treated as a dependent variable. For the creation of a model for artificial intelligence techniques, in particular for machine learning techniques as a branch of artificial intelligence, it is important that the created models perform well and calculate with good accuracy for a new series of data that have been unused so far. Therefore, the input data for the creation of the model is divided into two portions, which are often referred to as the train-test split (Salazar et al. 2022; Rashidi et al. 2019).

The first model learning technique used was linear regression. Linear regression is used to find a linear relationship between the target and one or more predictors. Currently, vehicle emission models are being developed using this method; examples include the works of Wang et al. (2022) and Madrazo and Clappier (2018).

The second method used was the random forest machine learning method. It is based on classification algorithms (Speiser et al. 2019). Random forest, as the name suggests, is a method based on so-called decision trees, which in combination form a random forest. The number of decision trees is very large, and they operate among themselves as an ensemble. Each individual tree in the random forest produces a class prediction, and the class with the model most votes becomes the prediction (Sheykhmousa et al. 2020; Schonlau and Zou 2020). In fact, a large number of relative tree models acting as an ensemble will outperform each of the individual component models in terms of prediction accuracy (Balyan et al. 2022). The reason for the good accuracy of this model is that trees protect themselves from making individual errors.

The third method used is gradient boosting. Boosting is a method to convert weak learners into strong learners (Bentéjac et al. 2021). In boosting, each new tree is a fit to a modified version of the original data set. The gradient boosting algorithm begins by training a decision tree in which each observation is assigned an equal weight. After evaluating the first tree, we increase the weights of the observations that are difficult to classify and decrease the weights of those that are easy to classify (Zhang et al. 2021b; Li et al. 2020). A second tree is then created on these weighted data. In this case, the idea is to improve the predictions of the first tree. Gradient boosting trains many models in an additive, sequential, and gradual manner. One of the main motivations for using gradient boosting is that it allows a user-specified cost function to be optimized, rather than a loss function, which typically offers less control and generally does not correspond to real-world applications (Punmiya and Choe 2019; Wang et al. 2020b).

**Fig. 7:**
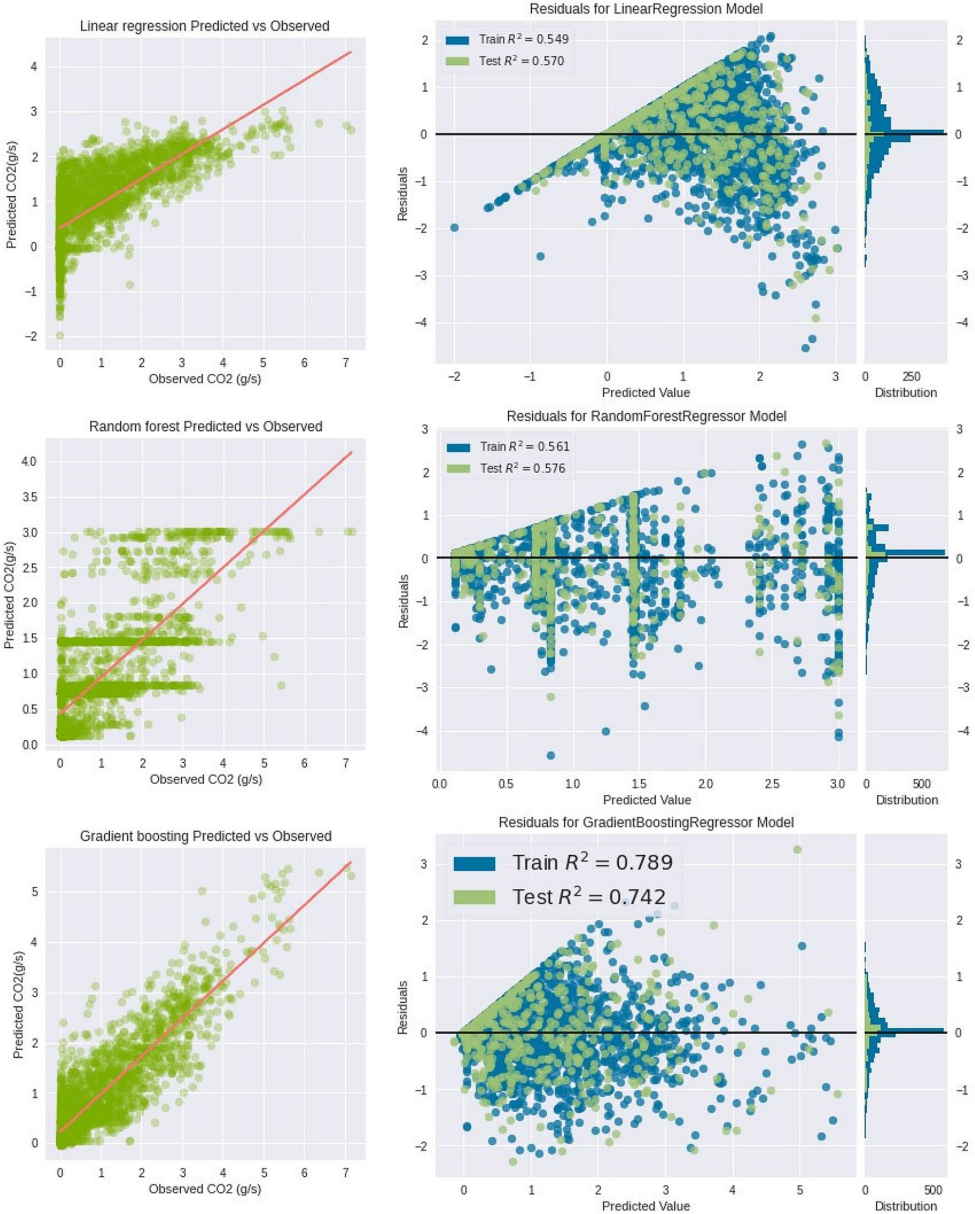
Scatter plot of observed versus predicted CO_2_ emission and residual plots for all machine learning methods

Figure 7 presents the results of the scatter plot of predicted versus observed CO_2_ emission and residual plots for all investigated machine learning methods. Statistical analysis of the results was carried out based on the work (Piñeiro et al. 2008). On the basis of the predicted versus observed graphs, it can be seen that the strongest correlation between the prediction data and the actual data is for the case of the gradient-boosting machine learning technique. The opposite situation applies to linear regression and the random forest technique. For the residuals plot, the distance that separates the prediction from the 0-value line and the symmetries of the created point cloud are evaluated. Positive residual values say that the average prediction was too low, while negative values mean that the prediction was too high, a value of 0 means that the prediction was perfect. Therefore, a closer localization near the 0 axis means that the created model is better able to reflect and calculate values close to the real ones (Kozak and Piepho 2018). In the case of the residuals graph, similarly to the predicted versus observed graphs, the best results are presented by the gradient boosting method. This state of affairs is justified by the most symmetrical distribution of results, which tends to form clusters near the center of the graph.

**Fig. 8:**
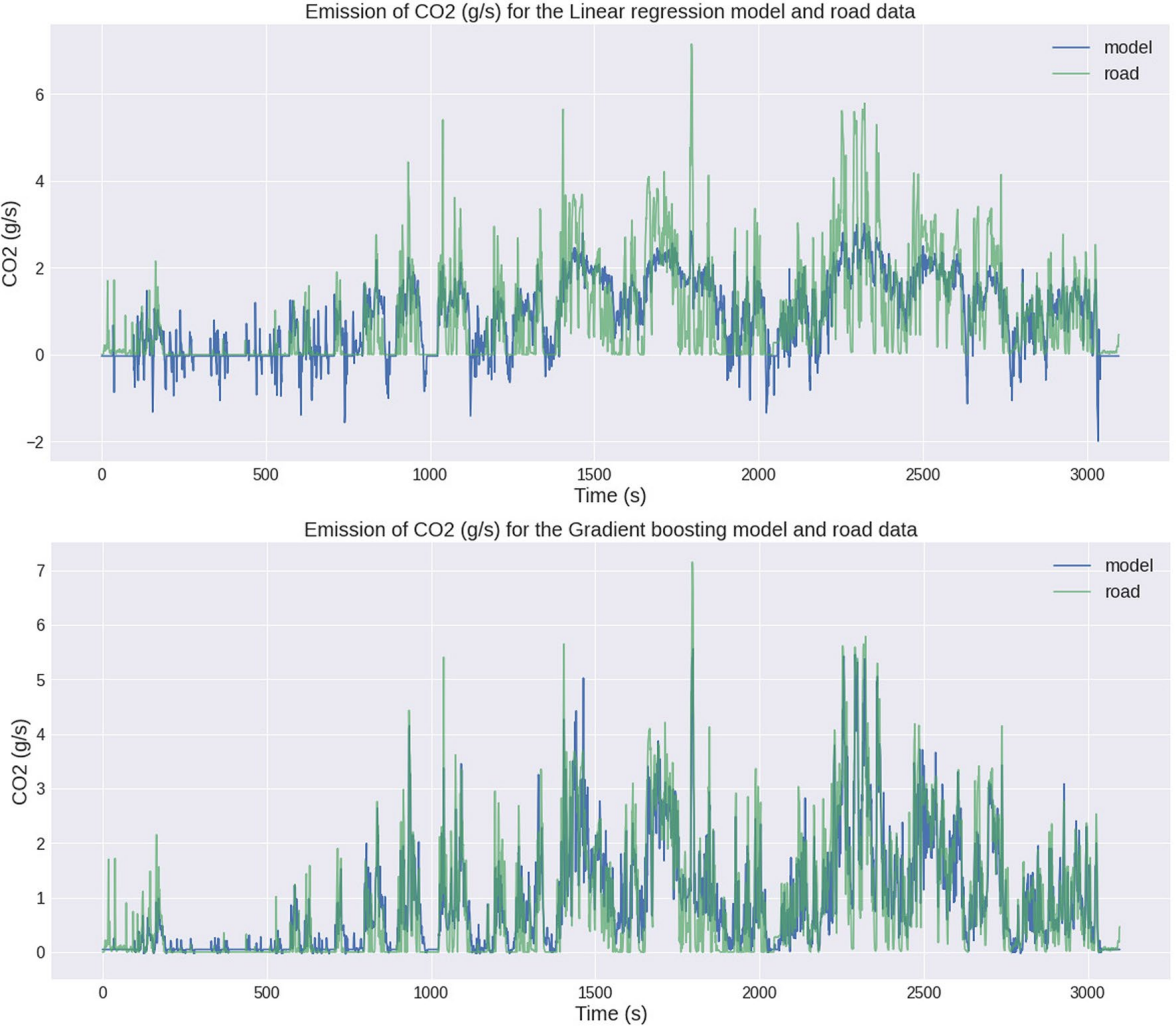
Instantaneous CO_2_ emission for linear regression and gradient boosting calculation method with comparison to road data

Figure 8 shows a plot of instantaneous CO_2_ emissions for real and model data for two selected methods of machine learning techniques: linear regression and gradient boosting. From this graph, we can also see how many times the vehicle has gone into start-stop procedure. The engine-off locations are periods where the CO_2_ emissions are 0 g/s. For the entire test route, 753 data records were found where the emission is 0 g/s. This means that the start-stop technology for the engine-off period was 12.55 min. This is a comparison of the methods and instantaneous emission results for the dataset studied. On the basis of this, it can be observed which method gives the best reflection of emission values, paying special attention to the zero emission values. These are, respectively, places where the vehicle is stopped and the engine is shut down, while not every machine learning technique is able to reflect such an engine operating condition. It can be seen from Fig. 8 that the linear regression method does not estimate CO_2_ emissions correctly, erroneously indicating even values below 0. Compared to this method, the gradient boosting method satisfactorily reflects the estimate of CO_2_ emissions, even for states where the engine is turned off. An area with greater differences in CO_2_ emissions is the state for a cold engine, where there was increased fuel consumption at the start of the road test, with consequent increased CO_2_ emissions. For practical use of the model and its simplicity, a separate model was not made for the engine state when it is characterized by the so-called cold start state.

**Fig. 9:**
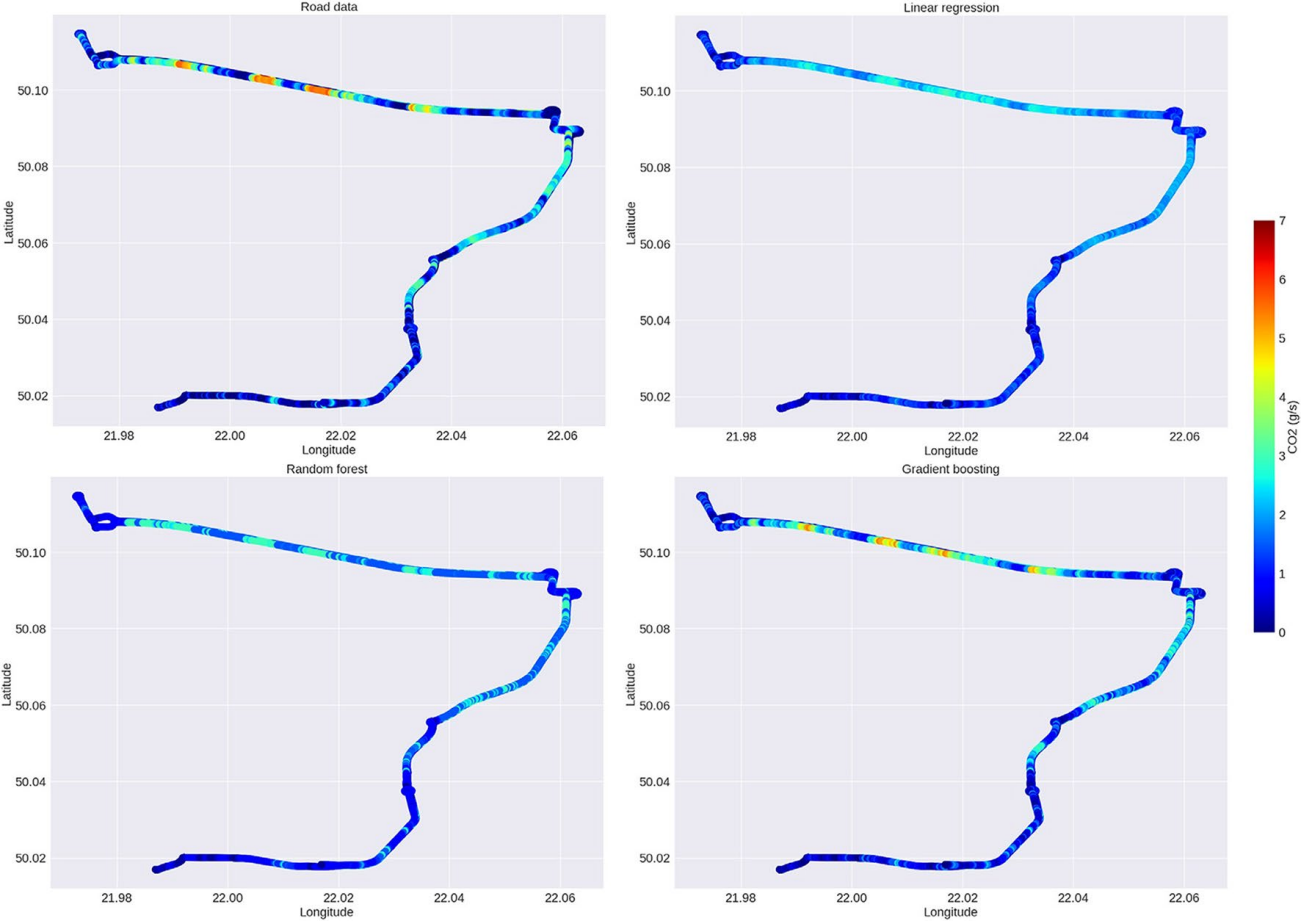
CO_2_ emission maps for the road data, linear regression, random forest, and gradient boosting prediction

Figure 9 shows the emission maps for the real-world data and the predictive data for all the machine learning techniques analyzed.

Based on the visualization of CO_2_ emission maps for start-stop technology, it is possible to see which model has the best predictive ability. For all the CO_2_ emission map data sets analyzed, the scale of values was unified to a range from 0 to 7 g/s. For example, the biggest differences for CO_2_ prediction indications are found for the linear regression method, which largely underestimates CO_2_ emissions, e.g., for a highway section, the emissions are relatively high over the course of the entire length of the highway compared to real data, where increased CO_2_ emissions occur only for selected areas of highway driving. A similar underestimation characterizes the random forest method. The map for the gradient boosting technique shows the closest to real values, which actually indicates very close values of CO_2_ emissions. This method has been shown to be very accurate both in terms of instantaneous emissions and CO_2_ emission maps for start-stop technology. It is also worth noting that, for the urban part, where there were many stopping points, this method performs a very accurate CO_2_ calculation, also indicating zero CO_2_ emission locations.

The validation of the models was also carried out using *R*^2^ and MSE. The coefficient of determination was calculated according to formula (2) (Ueki and Alzheimer’s Disease Neuroimaging Initiative 2021):

2 $${R}^{2}=\frac{{SS}_{M}}{{SS}_{T}}=\frac{\sum_{t=1}^{n}(\widehat{y}{ }_{t}-\overline{y }){ }^{2}}{\sum_{t=1}^{n}({y}_{t}-\overline{y }){ }^{2}}$$

where:

*R*^2^: coefficient of determination,
*SSM*: sum of squares for the model,
*SS*_*T*_: total sum of squares,
$$y_t$$ 
the actual value of the dependent variable,
$$\widehat{y}{ }_{t}$$ 
predicted values of the dependent variable,
$$\overline{y }$$ 
The average value of the actual dependent variable,
$$\sum_{t=1}^{n}$$ 
the sum of *k* = 1 to *n*.

The coefficient of determination describes how much variation in the explanatory variable was defined by the model (Chicco et al. 2021). It takes values in the range 0–1. Some sources state that the model fit is better the closer the *R*^2^ value is to unity, while this is incorrect, because the sheer number of observation data makes the model *R*^2^ value decrease; then, the evaluation should be carried out on the basis of other model validation parameters (Mohammad 2020). For the case under study, this involves analyzing instantaneous emission graphs and CO_2_ emission maps.

The evaluation of the model’s correctness is the most important part in the model development process because on this basis; it can be determined to what extent the prepared model fulfills its intended purpose. The validation of the obtained exhaust emission models was carried out on the basis of instantaneous and emission map results, using data that were not used for the calibration of earlier models. To validate the models obtained, a widely used coefficient was used to evaluate the prediction error, MSE. The mean squared error measures the amount of error in statistical models (Wang and Lu 2018; Gao et al. 2020) and assesses the average square difference between the observed and predicted values (Chani-Cahuana et al. 2018).

The mean square error was calculated based on the formula (Chachlakis et al. 2021) (3):

3 $$MSE=\frac{\sum \left(y-{y}_{t}^{P}\right)}{n}$$

where:

*y*: the actual value of the dependent variable,
$${y}_{t}^{P}$$ 
predicted values of the dependent variable,
*n*: number of observations.

The squaring of the differences in the MSE method serves several purposes. Squaring the differences eliminates negative values for the differences and ensures that the mean squared error is always greater than or equal to zero (Liu et al. 2018; Sun and Huang 2020). It is almost always a positive value. Only a perfect model without error produces an MSE of zero. Obtaining an MSE equal to zero is impossible in practice. The results shown in Table 3 confirm the previously inference methods for the analyzed to create a CO_2_ model using artificial intelligence methods. For both the training set and the test set, the gradient boosting method had the smallest prediction error rates. The same is true for *R*^2^, which for the gradient boosting method for the training set was 0.78, while for the test set, it was 0.74. These results indicate a very good representation of the real data for the model data. The worst method of those analyzed turned out to be the linear regression method, which achieved the weakest results for both the MSE and *R*^2^.

**Table 3:** MSE and *R*^2^ results for the training and test set

Method	Training MSE	Training *R*^2^	Test MSE	Test *R*^2^
Linear regression	0.611291	0.549273	0.592783	0.56976
Random forest	0.596003	0.560545	0.584536	0.575746
Gradient boosting	0.286721	0.78859	0.355065	0.742295

## Discussion

The developed CO_2_ emissions model for start-stop technology allows a more accurate representation of emissions, especially in urban traffic conditions, than commonly available emissions models. For example, the Versit + Enviver microscale model, developed by the Dutch research institute TNO, does not allow the analysis of this type of technology in vehicles (Quaassdorff et al. 2022; Severino et al. 2022). The VERSIT + speed profile-based emissions model, which is used in Enviver, is a multivariate regression model in which the driving cycle of a given vehicle is a variable (Anagnostopoulos and Kehagia 2018). It requires that speed profiles be obtained in advance, from which emission factors (g/km) can be estimated for different classes of vehicles (Chauhan et al. 2019). Another example of a model that also does not have an emission model specification for start-stop technology is the CMEM model. Based on power measurement, the CMEM model was developed in 2006. Emission processes are divided into different categories that correspond to physical phenomena associated with vehicle operation (Dong 2022). Each component of the exhaust gas is modeled analytically and includes parameters specific to its generation process. When developing these models, emissions were measured both directly in the engine and at the exhaust outlet of the vehicle under study (Kan et al. 2018a, b). The model requires detailed data, such as air resistance. Sometimes, however, parameters such as the air resistance value for the vehicle are not available. Therefore, instead of instantaneous power, this model uses vehicle-specific power (VSP), which is defined as the engine output per unit of vehicle mass and is expressed as a function of vehicle speed, road gradient, and acceleration (Perugu 2019; Acuto et al. 2022).

Other models of CO_2_ emissions are also available that are based on artificial intelligence techniques. An example is the model described in Mądziel et al. (2021). The authors of this work developed a CO_2_ emission model for a hybrid vehicle using the Gaussian process regression (GPR) machine learning technique. The model yielded *R*^2^ values of 0.69, while the MSE was 1.08. The velocity, acceleration, and road gradient parameters were also used for the input data to create the model. Another example of the use of machine learning techniques can be found in work (Jaworski et al. 2019), in which the authors, for vehicles that meet Euro 2-Euro 6 standards, created a special emissions model dedicated to the traffic characteristics of driving in roundabouts. The best method of machine learning techniques for this work turned out to be the boosted regression trees method, while validation of the model results was based on statistical analysis of coefficient of regression, root mean square error, and mean absolute error, and based on the visual assessment of the results, it was shown that the obtained models are well represented by real data. Another example is a novel approach to modeling machine learning emission to reduce vehicle brake emissions (Wei et al. 2022). The authors used a dataset of 600 real-world braking events. On the basis of the five algorithms, the importance of the different features of the fragments was discussed. Prediction performance was measured using *R*^2^ and RMSE. An interesting work in terms of emission modeling is work (Xu et al. 2020) where the authors proposed the deep learning reinforcement emission control strategy, which automatically learns the optimal traffic flow. Another work that presents a similar methodology for creating a CO_2_ emissions model is the work (Mądziel 2023a). This work shows the development of a novel methodology to create microscale CO_2_ emission models tailored specifically designed for LPG vehicles. The model is meticulously constructed, drawing on data collected from road tests utilizing the portable emission measurement system (PEMS) and the onboard diagnostic (OBDII) interface. Using advanced gradient-boosting machine learning techniques, this model was derived following an in-depth exploratory data analysis. Using vehicle velocity and engine RPM as key explanatory variables for a precise CO_2_ prediction. Rigorous validation of the model shows its precision, rendering it a valuable tool for continuous CO_2_ emission analysis and the creation of emission maps essential for environmental assessments in urban areas. The validation coefficients underscore its effectiveness, with an *R*^2^ test score of 0.61 and an MSE test score of 0.77. Another example would be a paper (Estrada et al. 2023) in which the authors use different input variables to create the model which limits its use for purposes such as simulating vehicle movement. This article introduces a novel approach to developing hybrid models designed to facilitate the testing of EMS and the assessment of fuel consumption, as well as CO_2_ and pollutant emissions (CO, NOx, and THC). In the context of this research, the authors highlight the limitations of static models like the widely-used map-based approach, which struggles to accurately quantify pollutant emissions due to the significant influence of transient effects. The primary innovation in this paper lies in the utilization of computational neural networks (CNN) to characterize pollutant emissions, thereby achieving high precision for both instantaneous and cumulative values. The input parameters used in this methodology are classical measurements associated with ICEs, which encompass engine speed, air mass flow, torque, and exhaust temperature. Another work that is a good and current example of the use of artificial intelligence methods for modeling CO_2_ emissions from vehicles is that of Mądziel (2023c), where this process is described for low emission zones in cities. In this research, a two-dimensional emission model for hybrid vehicles is introduced. This model utilizes artificial neural networks specifically designed for low-emission zones. The primary achievement of this study is the development of a tailored CO_2_ emission model for hybrid vehicles, enabling the simulation of different road solutions. Notably, the CO_2_ emission model exhibited a high *R*^2^ coefficient of 0.73 and a low MSE of 0.91. There are many methods for developing data for emission models, while it is important to choose the right one relative to the assumed expectations. This problem poses a major challenge for researchers, while a growing body of work contributes to better matching the appropriate methods with the expected predictive results. When it comes to selecting the right method, it is also useful to make use of compiled reviews of research works in the field of vehicle emissions modeling, an example is the work of (Mądziel 2023b).

From a review of the sources of other works, a gap emerged in the absence of a model specifically dedicated to vehicle equipped with start-stop technology. The model developed using artificial intelligence techniques addresses this need. Undoubtedly, this model can be used for a more accurate analysis of emission sources, especially in the urban part, where the highest traffic density actually occurs. A better representation of the emissions for such areas can undoubtedly contribute to a more accurate analysis of vehicle emissions and the impact of these emissions on the health of pedestrians or cyclists who travel along urban arteries or cross them at pedestrian crossings. Ecological analyses of vehicle emission sources are growing in importance every year. Microscale models can also be used to study the feasibility of investments in terms of ecological analysis. Emission maps obtained from the model can be helpful to decision-makers in city managements, in order to make better decisions for regional development of urban agglomerations taking into account environmental aspects.

## Conclusions

In this study, a methodology for creating a machine learning model for predicting CO_2_ emissions is presented for a test vehicle equipped with start-stop technology. Several possible machine learning techniques for CO_2_ prediction are presented, including linear regression, random forest, and gradient boosting methods, and the validation indices of these methods are indicated. The gradient boosting calculation method has the best predictive capability for CO_2_ emissions. The model validation indices for this method were determined separately for the training set (which represented 80% of the data used to build the model) and the test set (which consisted of 20% of previously unseen data). For the training set, the mean squared error (MSE) was 0.28, and the coefficient of determination (*R*^2^) was 0.78. In the case of the test set, the MSE was 0.35, and the *R*^2^ was 0.74.

The main conclusions of the work are:

- The computational technique of machine learning gradient boosting for the case studied had the best predictive capabilities for start-stop technology.
- The developed models could be used in the development of environmental analysis by, for example, local governments.
- The developed methodology may be scalable for the analysis of emissions from other vehicles with start-stop technology.
- The developed model is based on input parameters: velocity, vehicle acceleration, and road gradient. Therefore, it can also be used in simulation post-programming such as Vissim.

The limitations of the work are the development of the model based only on the data of one vehicle with start-stop technology. Although this is a preliminary model that will be expanded, the idea of the work was to select a machine learning technique that can reflect zero emissions during vehicle stops.

Future work includes continuous improvement of the model by measuring CO_2_ emissions for more vehicles. The work will specifically address the inclusion of hybrid vehicles in the test fleet. It is desirable to learn and test another pool of different artificial intelligence computing techniques that can be used to estimate CO_2_ emissions even more accurately under traffic conditions.

## Data Availability

The datasets used and/or analyzed during the current study are available from the corresponding author on reasonable request.

## Nomenclature

*a*: Vehicle acceleration (m/s^2^)
ANN: Artificial neural network
CLD: Chemiluminescence detector
CO: Carbon monoxide
CO_2_: Carbon dioxide
CPU: Central processing unit
CR: Common rail
DOC: Diesel oxidation catalysts
DT: Analyzer measurement delays
DPF: Diesel particulate filter
FID: Flame ionization detector
GPU: Graphics processing unit
GPR: Gaussian process regression
GRU: Gated recurrent unit
M: Molecular weight
MLP: Multilayer perceptron
MSE: Mean squared error
NDIR: Nondispersive infrared
NO_x_: Nitrogen oxides
OBD: On-board diagnostic
PEMS: Portable emission measurement system
*R*^2^: Coefficient of determination
SCR: Selective catalytic reduction
TPU: Tensor processing unit
*Q*: Gas flow rate
WLTP: Worldwide Harmonized Light-Duty Vehicles Test Procedure
*V*: Vehicle velocity (km/h)
